# Characterization of ^111^In-labeled Glucose-Dependent Insulinotropic Polypeptide as a Radiotracer for Neuroendocrine Tumors

**DOI:** 10.1038/s41598-018-21259-3

**Published:** 2018-02-13

**Authors:** Stefanie M. A. Willekens, Lieke Joosten, Otto C. Boerman, Maarten Brom, Martin Gotthardt

**Affiliations:** 1 Department of Radiology and Nuclear Medicine, Radboud university medical center, Nijmegen, The Netherlands; 2 Division of Nuclear Medicine and Molecular Imaging, Department of Imaging and Pathology, University Hospitals and KU Leuven, Leuven, Belgium

## Abstract

Somatostatin receptor targeting is considered the standard nuclear medicine technique for visualization of neuroendocrine tumors (NET). Since not all NETs over-express somatostatin receptors, the search for novel targets, visualizing these NETs, is ongoing. Many NETs, expressing low somatostatin receptor levels, express glucose-dependent insulinotropic polypeptide (GIP) receptors (GIPR). Here, we evaluated the performance of [Lys^37^(DTPA)]N-acetyl-GIP_1-42_, a newly synthesized GIP analogue to investigate whether NET imaging via GIPR targeting is feasible. Therefore, [Lys^37^(DTPA)]N-acetyl-GIP_1-42_ was radiolabeled with ^111^In with specific activity up to 1.2 TBq/µmol and both *in vitro* and *in vivo* receptor targeting properties were examined. *In vitro*, [Lys^37^(^111^In-DTPA)]N-acetyl-GIP_1-42_ showed receptor-mediated binding to BHK-GIPR positive cells, NES2Y cells and isolated islets. *In vivo*, both NES2Y and GIPR-transfected BHK tumors were visualized on SPECT/CT. Furthermore, co-administration of an excess unlabeled GIP_1-42_ lowered tracer uptake from 0.7 ± 0.2%ID/g to 0.6 ± 0.01%ID/g (p = 0.78) in NES2Y tumors and significantly lowered tracer uptake from 3.3 ± 0.8 to 0.8 ± 0.2%ID/g (p = 0.0001) in GIPR-transfected BHK tumors. In conclusion, [Lys^37^(^111^In-DTPA)]N-acetyl-GIP_1-42_ shows receptor-mediated binding in various models. Furthermore, both GIPR-transfected BHK tumors and NES2Y tumors were visible on SPECT/CT using this tracer. Therefore, [Lys^37^(^111^In-DTPA)]N-acetyl-GIP_1-42_ SPECT seems promising for visualization of somatostatin receptor negative NETs.

## Introduction

Targeting of peptide hormone receptors, expressed on tumors, is a valuable tool for *in vivo* molecular imaging and radionuclide therapy of a variety of neuroendocrine tumors (NET). Successful tumor targeting relies on over-expression of target receptors on cancer cells when compared to their expression levels in healthy tissues^1,2^. Nowadays, somatostatin receptor targeting is a standard procedure for NET detection^3–6^ and radionuclide therapy with ^90^Y- and ^177^Lu-labeled somatostatin analogues was proven beneficial to patients with NET^7^. However, not all NETs show elevated somatostatin receptor expression levels. Therefore, false negative results may occur in all NET types and somatostatin receptor imaging displays limited sensitivity for some types of NET, such as insulinomas (80–90% on ^68^Ga-DOTATATE PET)^3,5,8–10^ and medullary thyroid carcinoma (MTC) for which the detection rate is below 40–60%^11^.

A variety of alternative peptide receptors, such as cholecystokinin (CCK) receptors^12^, vasoactive intestinal peptide (VIP) receptors^13^ and glucagon-like peptide 1 (GLP-1) receptors (GLP-1R)^14^ are expressed on NET. Recent findings showed glucose-dependent insulinotropic polypeptide (GIP) receptor (GIPR) expression in gastric, duodenal and bronchial NET^15,16^. Furthermore, approximately 90% of somatostatin receptor negative NET are GIPR positive^16^. In 2013, Sherman *et al*. pointed out that GIPR is a promising target for NET imaging and therapy due to its favorable expression pattern^17^ and the feasibility of GIPR targeting was shown in a GIPR over-expressing tumor model using a truncated GIP_1-30_ analogue^18^.

GIP is secreted from the intestinal K cells after nutrient ingestion^19^ and together with GLP-1, it enhances glucose-induced insulin secretion upon receptor binding on pancreatic beta cells, the so-called incretin effect^20,21^. Since insulinomas are NETs derived from pancreatic beta cells, incretin receptors are expected to be ideal candidate receptors for insulinoma imaging. Indeed, GLP-1R targeting tracers, such as [^68^Ga]- or [^111^In]-labeled exendin, can be applied for non-invasive *in vivo* insulinoma detection^22–24^. However, since malignant insulinomas display differential GLP-1R and somatostatin receptor expression patterns^25^, detection rates of these tumors remain limited to 50% by scintigraphy. Interestingly, GLP-1R negative malignant insulinomas and a majority of somatostatin negative NETs express enhanced GIPR levels^16^, rendering this receptor an interesting target for NET and insulinoma imaging.

**Figure 1:**
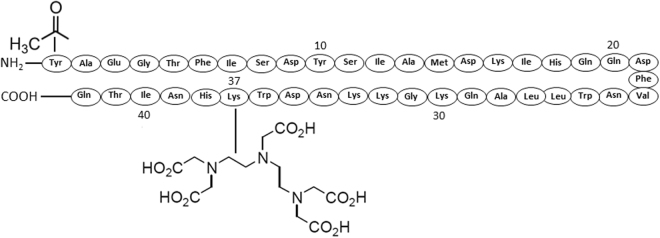
Amino acid sequence and molecular modifications of [Lys^37^(^111^In-DTPA)]N-acetyl-GIP_1-42_.

Since it was described that GIP_1-30_ exhibits reduced receptor binding affinity when compared to the full length peptide GIP_1-42_^26,27^, we have investigated the potential of a newly synthesized GIP_1-42_ analogue, [Lys^37^(^111^In-DTPA)]N-acetyl-GIP_1-42_ (Fig. 1) as a radiotracer for NET imaging, starting from the initial hypothesis that a full-length peptide-based tracer might show improved characteristics for *in vivo* NET imaging when compared to GIP_1-30_. Therefore, we optimized the radiolabeling procedure and investigated its binding and internalization kinetics using GIPR-positive tumor cells (BHK-GIPR). Furthermore, we have also explored the tracers binding characteristics to NES2Y cells (a human beta cell-derived cell line, representing a more realistic model in terms of receptor expression)^28^ and isolated islets of Langerhans. Finally, subcutaneous BHK-GIPR and NES2Y tumors were visualized by SPECT after injection of [Lys^37^(^111^In-DTPA)]N-acetyl-GIP_1-42_.

## Results

### Radiolabeling and Serum Stability

**Figure 2:**
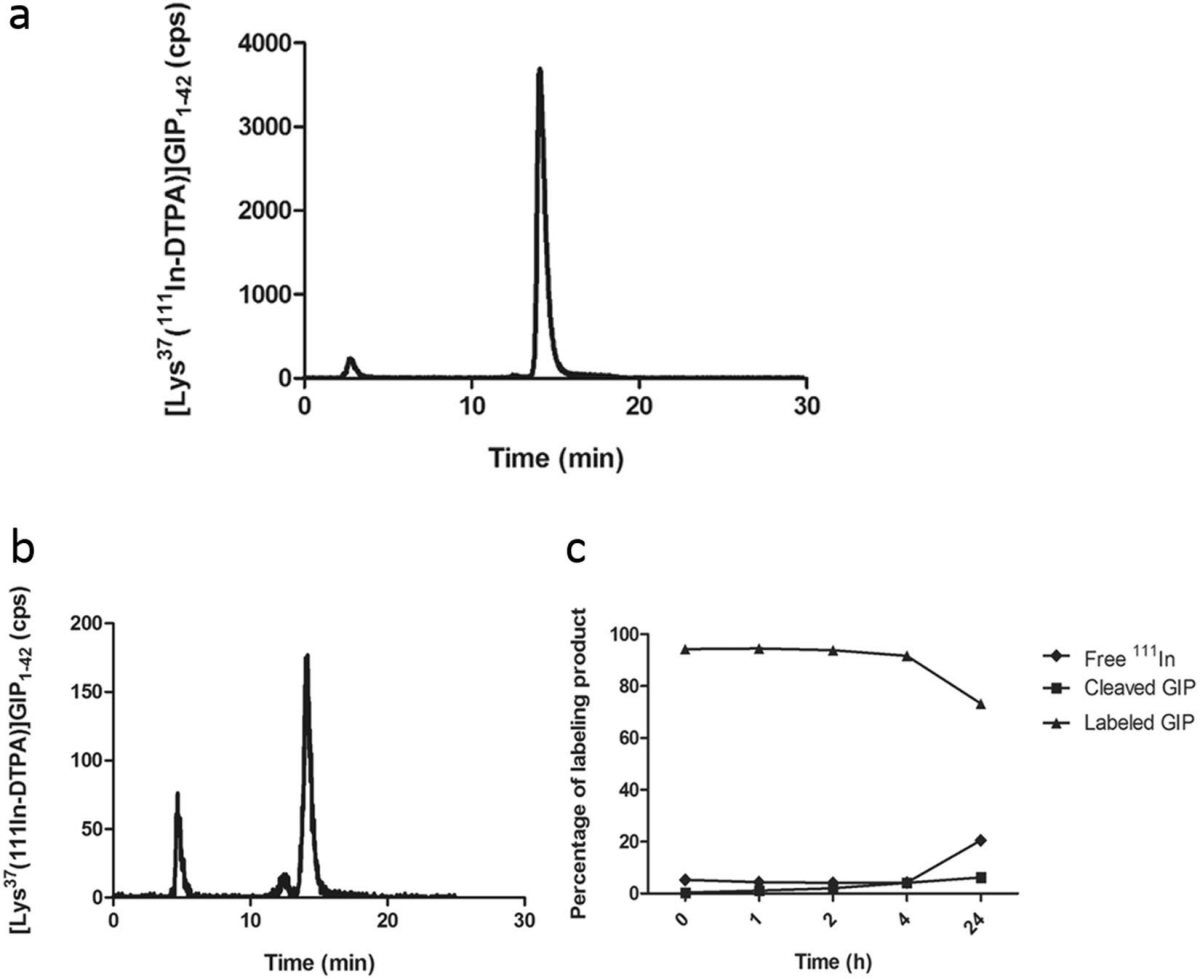
(**a**) Radiochemical purity analysis of [Lys^37^(^111^In-DTPA)]N-acetyl-GIP_1-42_ as determined by HPLC, immediately after labeling. (**b**) HPLC profile of ^111^In-GIP after 24 hours of incubation in human serum. (**c**) Overview of stability behavior after various incubation times in human serum.

[Lys^37^(DTPA)]N-acetyl-GIP_1-42_ could be labeled with ^111^In with a specific activity (now referred to as molar activity^29^) of up to 1.2 TBq/µmol. Radiochemical purity exceeded 95% as determined by RP-HPLC and ITLC, resulting in a final molar activity exceeding 142.5 MBq/µg or 712.5MBq/nmol when starting with 150 MBq [^111^In]Cl_3_. Figure 2a shows the HPLC analysis of the labeling mixture. ^111^In-EDTA eluted with a retention time of 3 minutes, whereas ^111^In labeled [Lys^37^(DTPA)]N-acetyl-GIP_1-42_ had a retention time of 14 minutes. After 12 minutes, a very small impurity (<2%) eluted from the column. Since GIP is known to be prone to inactivation by dipeptidyl peptidase IV (DPP IV), the stability of [Lys^37^(^111^In-DTPA)]N-acetyl-GIP_1-42_ was analyzed in human serum. The results of this stability analysis are shown in Fig. 2b and c. Up to 4 hours of incubation in human serum, the [Lys^37^(^111^In-DTPA)]N-acetyl-GIP_1-42_ remained intact. After 24 hours of incubation in human serum, 73% of the activity was still found as intact radiolabeled peptide, as determined by HPLC.

### *In vitro* binding and internalization kinetics

**Figure 3:**
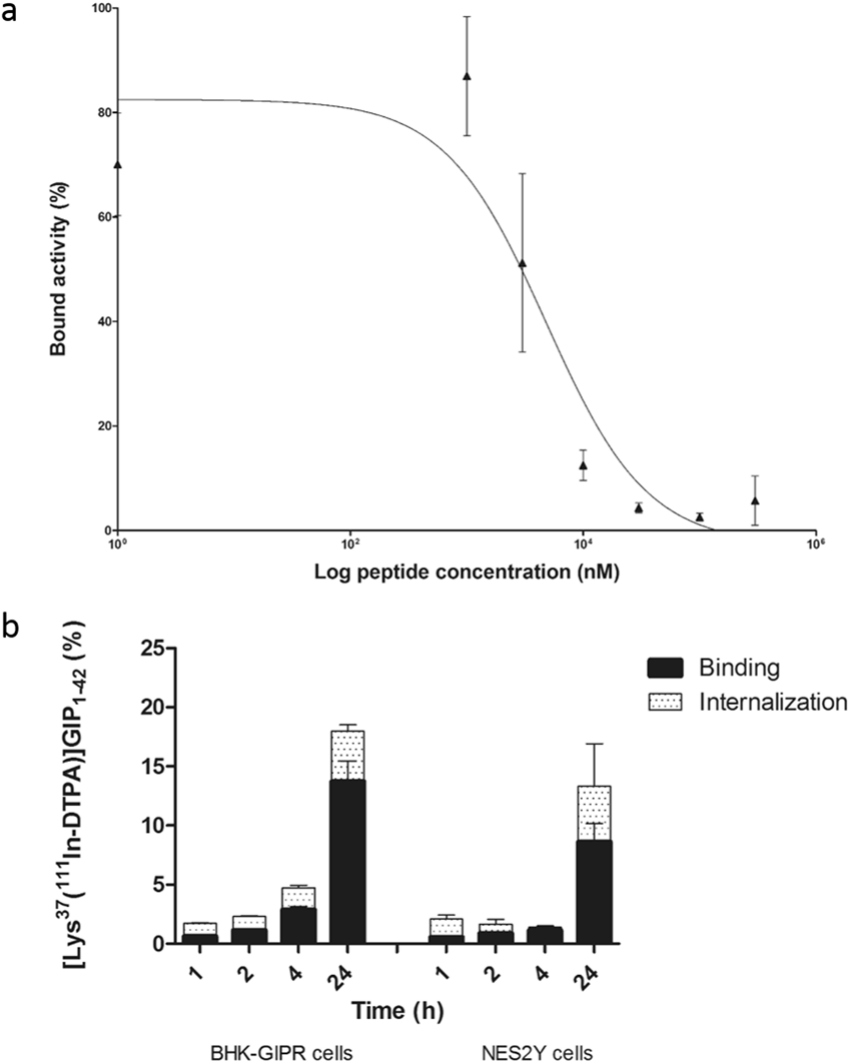
*In vitro* characterization (**a**) Competition binding assay (apparent IC_50_) of [Lys^37^(DTPA)]GIP_1-42_ on NES2Y cells. [Lys^37^(^111^In-DTPA)]N-acetyl-GIP_1-42_ was used as a radiotracer (**b**) binding and internalization kinetics of ^111^In-GIP in BHK-GIPR positive cells and NES2Y cells. Cell bound and internalized fractions are corrected for non-specific binding and accumulation, as determined by co-incubation with an excess unlabeled GIP_1-42_.

**Figure 4:**
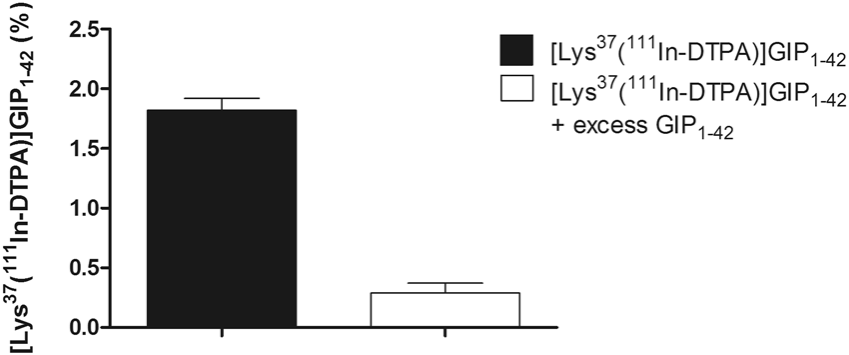
Binding of [Lys^37^(^111^In-DTPA)]N-acetyl-GIP_1-42_ to isolated islets. The solid bar represents binding of [Lys^37^(^111^In-DTPA)]N-acetyl-GIP_1-42_. The white bar represents binding of [Lys^37^(^111^In-DTPA)]N-acetyl-GIP_1-42_ in the presence of an excess GIP_1-42_.

The results of the apparent IC_50_ determination are shown in Fig. 3a. The apparent IC_50_ of [Lys^37^(^111^In-DTPA)]N-acetyl-GIP_1-42_ is 4.8 µ*M* (95% confidence interval: 0.7–32.8 µ*M*). However, the low internalization rate described below, render the observed value more likely to be a true IC_50_ rather than an apparent IC_50_. Figure 3b summarizes the binding and internalization kinetics of [Lys^37^(^111^In-DTPA)]N-acetyl-GIP_1-42_ by BHK-GIPR transfected cells and NES2Y cells as determined *in vitro*. Both cell lines displayed similar binding and internalization characteristics. After 1 hour, 0.6 ± 0.1% and 0.6 ± 0.02% of the added [Lys^37^(^111^In-DTPA)]N-acetyl-GIP_1-42_ was bound to BHK-GIPR transfected cells and NES2Y cells, respectively. At this time point, the internalized fractions were 1.1 ± 0.1% and 1.5 ± 0.3%, respectively. After 24 hours, the binding values had increased to 13.8 ± 1.7% and 8.6 ± 1.5%, respectively and the internalization values to 4.2 ± 0.6% and 4.7 ± 3.6% for BHK-GIPR and NES2Y cells, respectively. All values were corrected for non-specific binding, as determined by the addition of 25 µg unlabeled GIP_1-42_ which significantly reduced the binding and internalization at all time points indicating GIPR-mediated binding in both cell lines (p < 0.05). Figure 4 shows the *in vitro* binding characteristics of [Lys^37^(^111^In-DTPA)]N-acetyl-GIP_1-42_ to 200 isolated islets of C3H mice. After 24 hours, 0.22 ± 0.01 fmol [Lys^37^(DTPA)]GIP_1-42_ bound to the islets. Addition of 25 µg unlabeled GIP_1-42_ reduced the binding to 0.03 ± 0.01 fmol, indicating specific binding of the tracer to the GIPR on the islets of Langerhans.

### Peptide Dose Escalation Study

**Figure 5:**
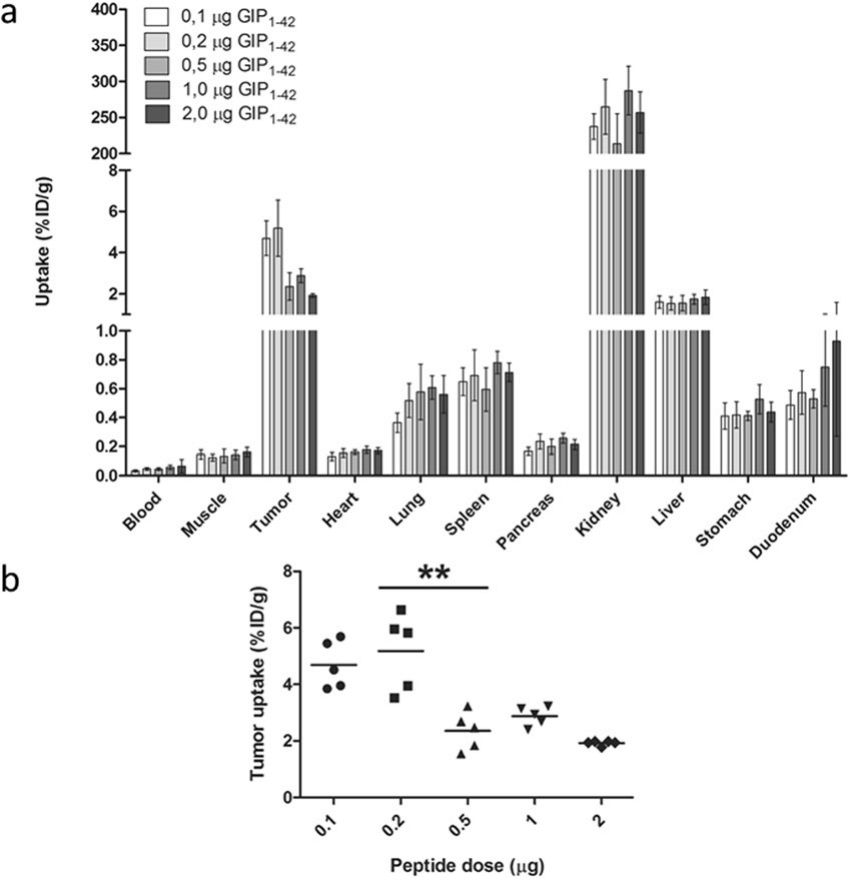
(**a**) Peptide dose escalation study in BALB/c nude mice with subcutaneous BHK-GIPR tumors. Values are expressed as percentage injected dose per gram tissue (%ID/g) (n = 5). (**b**) Tumor uptake of [Lys^37^(^111^In-DTPA)]N-acetyl-GIP_1-42_ using different peptide doses. Tumor uptake was significantly higher (**p = 0.008) at peptide doses smaller than 0.5 µg (0.1 nmol).

The effect of the peptide dose on tumor targeting was studied in BALB/c nude mice with subcutaneous BHK-GIPR tumors and the results are summarized in Fig. 5a. The [Lys^37^(DTPA)]N-acetyl-GIP_1-42_ dose had a pronounced effect on tumor uptake, which decreased significantly at peptide doses exceeding 0.2 µg (Fig. 5a and b). Highest tumor uptake of [Lys^37^(^111^In-DTPA)]N-acetyl-GIP_1-42_ was observed at peptide doses ≤ 0.2 µg (0.04 nmol) per mouse: 5.2 ± 1.4%ID/g at 4 hours post-injection. Administration of 0.5 µg (0.1 nmol) as a peptide dose resulted in a significantly lower tumor uptake: 2.4 ± 0.7%ID/g (p = 0.008) (Fig. 5b). Four hours post-injection, [Lys^37^(^111^In-DTPA)]N-acetyl)GIP_1-42_ also showed uptake in various organs such as the lung, spleen, pancreas, liver, stomach and duodenum. The high uptake in the kidneys has been described as being the result of tubular reabsorption through the scavenger receptors cubilin and megalin, which is the case for many peptides^30^. As the kidney uptake of radiolabeled Lys^37^(^111^In-DTPA)]N-acetyl-GIP_1-42_ cannot be inhibited by an excess of unlabeled peptide, tubular reabsorption indeed appears to be the cause of the high kidney uptake. Raw data of this biodistribution study are shown in supplementary Table 1.

### Biodistribution Studies

**Figure 6:**
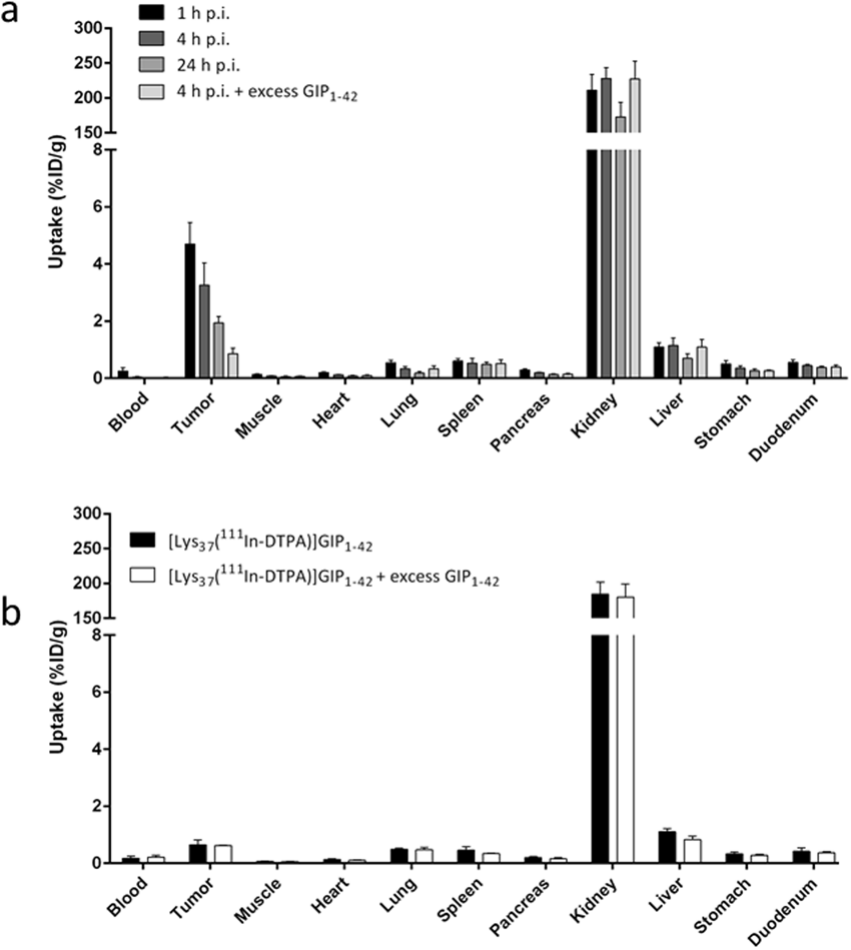
(**a**) Biodistribution study of [Lys^37^(^111^In-DTPA)]N-acetyl-GIP_1-42_ in BALB/c nude mice bearing subcutaneous BHK-GIPR tumors at 1, 4 and 24 hours after tracer injection. Values are expressed as percentage injected dose per gram tissue (%ID/g) (n = 5). Blocking was performed by co-injected of 25 µg unlabeled GIP_1-42_ (n = 5). (**b**) Biodistribution study of [Lys^37^(^111^In-DTPA)]N-acetyl-GIP_1-42_ in BALB/c nude mice bearing subcutaneous NES2Y tumors, 1 hour after tracer injection. Values are expressed as percentage injected dose per gram tissue (%ID/g) (n = 5). Blocking was performed by co-injection of 25 µg unlabeled GIP_1-42_ (n = 2).

Figure 6a summarizes the biodistribution of [Lys^37^(^111^In-DTPA)]N-acetyl-GIP_1-42_ in BALB/c nude mice bearing a subcutaneous BHK-GIPR transfected tumor. One hour after injection, highest tumor uptake was observed: 4.7 ± 0.8%ID/g. After 4 and 24 hours, tumor uptake decreased to 3.3 ± 0.8%ID/g and 2.0 ± 0.2%ID/g, respectively (Fig. 6a). Co-administration of an excess unlabeled GIP_1-42_ significantly lowered tumor uptake to 0.8 ± 0.2%ID/g (p = 0.0001) 4 hours after injection, indicating GIPR-mediated tumor uptake. One hour after injection, tumor uptake in NES2Y tumors was 0.7 ± 0.2%ID/g (Fig. 6b). Co-administration of an excess unlabeled GIP_1-42_ lowered tumor uptake to 0.6 ± 0.01%ID/g (p = 0.78) Raw data of these biodistribution studies are shown in supplementary Tables 2 and 3.

### SPECT/CT imaging

**Figure 7:**
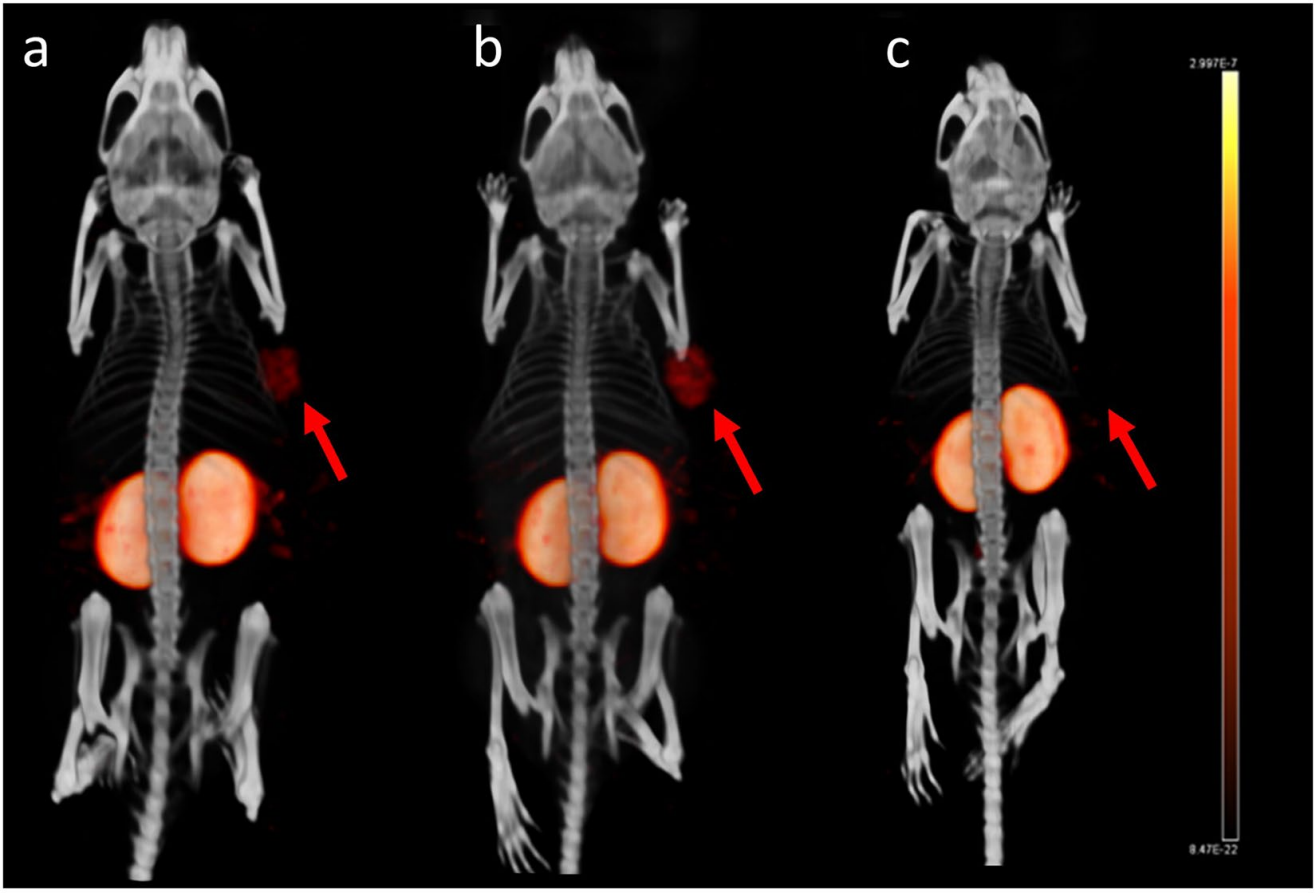
Maximum intensity projection (MIP) SPECT/CT images of a BALB/c nude mouse bearing a subcutaneous BHK-GIPR tumor 1 hour after injection (**a**), 4 hours after injection (**b**) and 4 hours after injection and co-injection with 25 µg unlabeled GIP_1-42_ (**c**).

**Figure 8:**
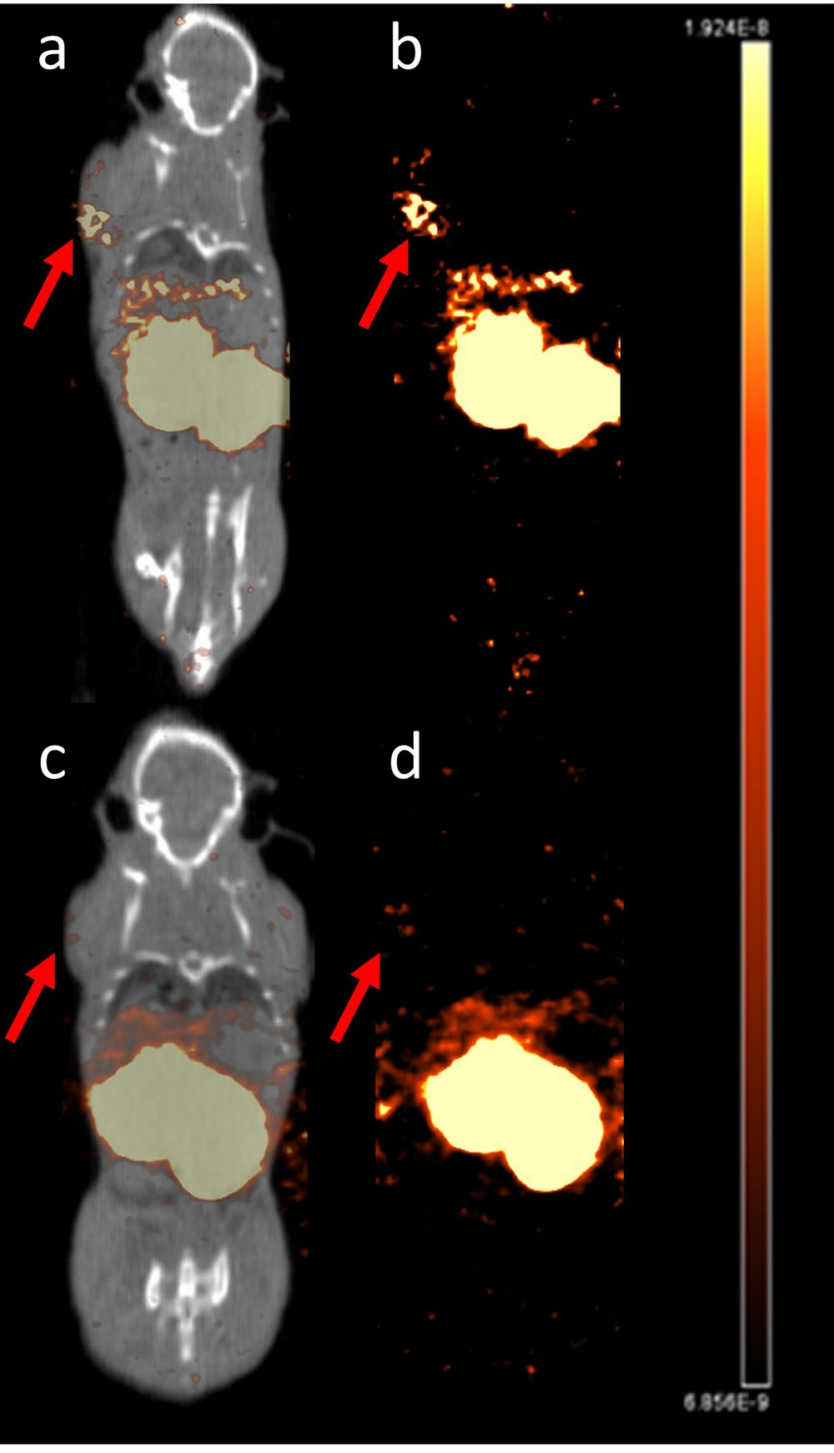
SPECT/CT (**a**) and SPECT (**b**) of a BALB/c nude mouse bearing a subcutaneous NES2Y tumor, 1 hour after injection and SPECT/CT (**c)** and SPECT (**d**) after co-injection with 25 µg unlabeled GIP_1-42_.

One and four hours after injection of [Lys^37^(^111^In-DTPA)]N-acetyl-GIP_1-42_, BHK-GIPR transfected tumors were clearly visible on SPECT/CT (Fig. 7a and b). Co-injection of an excess unlabeled GIP_1-42_ completely blocked tracer uptake in the BHK-GIPR transfected tumor (Fig. 7c). Also in mice bearing NES2Y tumors, clear tumor uptake was observed 1 hour after tracer injection (Fig. 8a and b). Furthermore, co-injection of an excess unlabeled GIP_1-42_ prevented tracer uptake in NES2Y tumors, suggesting GIPR-mediated tracer uptake in these tumors.

## Discussion

In this study, we investigated the potential of [Lys^37^(^111^In-DTPA)]N-acetyl-GIP_1-42_ for NET detection. The tracer showed GIPR-mediated binding to BHK-GIPR positive cells and NES2Y cells, and to isolated islets *in vitro*. Optimal GIPR-mediated tumor targeting of BHK-GIPR positive tumors *in vivo*, was observed 1 hour post injection using a peptide dose of 0.2 µg (0.04 nmol). Furthermore, both BHK-GIPR transfected tumors and NES2Y tumors were visualized by SPECT.

It has been described that C-terminally truncated GIP analogues, such as GIP_1-30_, exhibit reduced receptor binding affinity when compared to intact GIP_1-42_, while retaining their full insulinotropic effect^26,27^. Since the goal of this study was to apply the analogue for *in vivo* imaging, high receptor affinity should be preserved rather than biological activity. Therefore, we selected GIP_1-42_ as a basis for our radiotracer. Another point of consideration while designing a GIPR targeting radiotracer, is the susceptibility of the native peptide to DPP IV degradation^31^. Since it was shown that several N-terminal modifications showed increased half-life *in vitro*^32,33^, we acetylated the N-terminal tyrosine residue to obtain a better DPP IV degradation-resistant tracer. Indeed, we observed that the tracer remained stable in serum for at least 4 hours after incubation and even after 24 hours of incubation 73% of the tracer remained intact.

In order to obtain high quality images, high target-to-background ratios are required. Tracer metabolites labeled with a radiometal via a chelator, can be trapped in the lysosomes upon tracer internalization and degradation, resulting in enhanced tracer accumulation in the target cells^23,34^. Therefore, we investigated tracer internalization rates *in vitro*. Internalization kinetics of the tracer were comparable and relatively slow: after 24 hours, both BHK-GIPR positive and NES2Y cell showed similar internalization rates of 4.2 ± 0.6%ID/g and 4.7 ± 3.6%ID/g, respectively. Interestingly, the majority of the tracer was bound to the cell surface in both cell lines. These observations could be explained by very recent findings of Ismail *et al*. indicating that N-terminal acetylation of the peptide hampers receptor internalization^35^.

In BHK-GIPR positive tumors, GIP_1-42_ doses lower than 0.5 µg (0.1 nmol) resulted in maximal tumor accumulation. This indicates that administration of higher peptide doses results in partial saturation of the GIPR *in vivo*. However, since the tracer could be labeled with a specific activity up to 1.2 TBq/µmol, administration of an activity dose for imaging dose is still feasible. For *in vivo* SPECT imaging, high tumor-to-blood ratios are required. However, since tracer uptake in the blood was still very low at a peptide dose of 0.2 µg, we selected this dose as the optimal dose for *in vivo* imaging since it resulted in the highest absolute tumor uptake.

Although [Lys^37^(^111^In-DTPA)]N-acetyl-GIP_1-42_ showed high and specific tumor uptake in GIPR-transfected GIPR tumors, tumor uptake in NES2Y tumors was lower and co-injection of an excess unlabeled GIP_1-42_ did not result in significantly lower tracer uptake. This clear difference in tracer uptake might be explained by the completely different origin and purpose of both cell lines. While the BHK-GIPR cell line was created to express artificially high receptor levels, the NES2Y cells are derived from patients with congenital hyperinsulinism of infancy (28). Since these cells do not overexpress the target receptor and it is known that the natural expression levels of the GIPR are much lower compared to its brother incretin peptide GLP-1^36^, the lower uptake is to be expected. As for the blocking study, the small size of the blocking group (n = 2) impairs generation of representative results. Therefore, the obtained significance level (p = 0.78) might be distorted. On the SPECT images however, tracer accumulation was observed in NES2Y tumors but was no longer observed after co-injection of an excess unlabeled GIP_1-42_ (Fig. 8b), suggesting specific tumor targeting. Nevertheless, several tracer characteristics might be optimized to further improve tumor targeting. Firstly, other N-terminal modifications than acetylation, such as glucitol or pyroglutamil insertion, have been shown to improve the half-life of the peptide in the presence of DPP IV^32,33^ and might also result in higher internalization rates. Secondly, receptor binding and internalization might increase with longer circulation times. As shown in our biodistribution studies, [Lys^37^(^111^In-DTPA)]N-acetyl-GIP_1-42_ is cleared from the blood very rapidly, hampering longer tracer circulation and thus higher tumor uptake. Several methods such as PEGylation, multimerization or introduction of free fatty acid tails might increase circulation time^37^ and thereby tracer accumulation in the tumor. However, a good balance between tracer retention in blood and tracer targeting dynamics has to be found in order to obtain favorable target-to-background ratios for *in vivo* imaging.

[Lys^37^(^111^In-DTPA)]N-acetyl-GIP_1-42_ was produced at a high molar activity and showed receptor mediated binding to various GIPR expressing cells *in vitro*. Subcutaneous BHK-GIPR positive tumors showed GIPR-mediated tracer accumulation with optimal *in vivo* targeting 1 hour post-injection, at a peptide dose of 0.2 µg (0.04 nmol). Furthermore, although very low, uptake of a GIP-based tracer was demonstrated for the first time in a human beta-cell derived tumor. Unfortunately, specificity of tracer binding could not be shown due to the small size of the blocking group. In conclusion, [Lys^37^(^111^In-DTPA)]N-acetyl-GIP_1-42_ shows promising results as a radiotracer for NET imaging. However, the initial working hypothesis that a full-length peptide-based tracer might show improved characteristics for *in vivo* NET imaging when compared to GIP_1-30_ could not be confirmed.

## Methods

### Radiolabeling

[Lys^37^(DTPA)]N-acetyl-GIP_1-42_ (MW = 5422 g/mol) and GIP_1-42_ (MW = 5046.7 g/mol) were purchased from Peptide Specialty Laboratories (PSL GmbH, Heidelberg, Germany). DTPA was conjugated to the ε-amino group of the Lysine (K37) and the N-terminal tyrosine was acetylated to reduce its susceptibility to endopeptidases (Fig. 1). [Lys^37^(DTPA)]GIP_1-42_ was dissolved in 0.5 *M* MES (2-(N-morpholino)ethanesulfonic acid) buffer, pH 5.5 and stored at 4 °C until use. 150 MBq [^111^In]Cl_3_ (Mallinckrodt Pharmaceuticals, ‘s Hertogenbosch, The Netherlands) was added to 1 µg [Lys^37^(DTPA)]N-acetyl-GIP_1-42_ together with 5 volumes of 0.5 *M* MES buffer (1:5 relation ^111^In:buffer), pH 5.5 and incubated for 20 min at room temperature (RT). After incubation, EDTA (Sigma Aldrich, St. Louis, MO, USA) and Tween80 (Sigma Aldrich) were added to a final concentration of 5 m*M* and 0.1%, respectively. The labeling mixture was purified by solid-phase extraction using a hydrophilic-lipophilic balance (HLB) cartridge (30 mg, Waters Oasis, Milford, MA, USA), to eliminate unincorporated ^111^In. The cartridge was activated with 1 ml ethanol, washed with 2 ml water and conditioned with 1 ml of 0.5 *M* MES, pH 5.5. Subsequently, the cartridge was loaded with the labeling mixture, washed with 1 ml 0.5 *M* MES and 2 ml water and [Lys^37^(^111^In-DTPA)]N-acetyl-GIP_1-42_ was eluted from the cartridge with 200 µL 100% ethanol. For *in vivo* use, purified labeled peptide was diluted in phosphate buffered saline (PBS), 0.5% bovine serum albumin (BSA) (w/v) to obtain a final ethanol concentration of less than 10%. Radiochemical purity of [Lys^37^(^111^In-DTPA)]N-acetyl-GIP_1-42_ was determined by instant thin-layer chromatography (ITLC) (ITLC-SG, Agilent Technologies, Lake Forest, CA, USA), using 0.1 *M* EDTA in 0.1 *M* NH_4_Ac (Sigma Aldrich), pH 5.5 as a mobile agent and by reversed-phase high performance liquid chromatography (RP-HPLC) using an Eclipse XDB C18 column (Agilent Technologies). The column was eluted with 0.1% trifluoroacetic acid (TFA) in H_2_O (0-5 minutes) followed by a linear gradient from 3% to 100% acetonitrile with 0.1% TFA over 10 minutes (flow rate: 1 ml/minute).

### Serum Stability Analysis

For the serum stability analysis, [Lys^37^(DTPA)]N-acetyl-GIP_1-42_ was labeled with [^111^In]Cl_3_ at a specific activity of 27 GBq/µmol. Radiochemical purity of [Lys^37^(^111^In-DTPA)]N-acetyl-GIP_1-42_ directly after labeling was determined by ITLC-SG (Agilent Technologies) and RP-HPLC as described above. A sample of the labeling mixture was incubated in human serum (1:10) at 37 °C. After 1, 2, 4 and 24 hours, a sample was taken and serum proteins were precipitated by adding acetonitrile. This mixture was centrifuged and after dilution, the radiochemical purity of the compound in the supernatant was analyzed using ITLC and RP-HPLC as described above.

### Cell Culture

Baby hamster kidney cells, transfected with the human GIP (BHK-GIPR cells) were maintained in DMEM Glutamax (cat. Nr. 6195, GIBCO, BRL Life Sciences Technologies, Bleiswijk, The Netherlands) supplemented with 10% fetal bovine serum (FCS), (HyClone, Celbio, Logan, UT, USA) and 1 mg/ml G418, in a humidified atmosphere containing 5% CO_2_ at 37 °C. The human beta cell line NES2Y was cultured as described previously (28). Briefly, cells were maintained in RPMI 1640 (R0883, GIBCO) supplemented with 10% FCS, (HyClone, Celbio), 2 m*M* L-glutamine (Sigma Aldrich), 100 U/ml penicillin (Sigma Aldrich) and 100 µg/ml streptomycin (Sigma Aldrich) (complete RPMI), in a humidified atmosphere containing 5% CO_2_ at 37 °C.

### IC_50_ Determination

The 50% inhibitory concentration (IC_50_) of [Lys^37^(^111^In-DTPA)]N-acetyl-GIP_1-42_ was determined using NES2Y cells. NES2Y cells were seeded at a density of 200,000 cells/well in 24-well plates and incubated overnight at 37 °C. The cells were washed with binding buffer (RPMI + 0.5% BSA (w/v)) and [Lys^37^(DTPA)]N-acetyl-GIP_1-42_ was added at a final concentration ranging from 1.0 – 100 µ*M* along with radiolabeled [Lys^37^(^111^In-DTPA)]N-acetyl-GIP_1-42_ (4.6 kBq = 2.7 fmol). After o/n (15 h) incubation at 37 °C, cells were washed with binding buffer and cell-associated activity was measured in a well type gamma counter (Wallac 2480 wizard, Perkin Elmer). Under these conditions, internalization may occur. We therefore designate the results of this competitive binding assay as “apparent IC_50_” values rather than IC_50_.

### Internalization kinetics

The internalization kinetics of [Lys^37^(^111^In-DTPA)]N-acetyl-GIP_1-42_ was determined as previously described^38^ using BHK-GIPR transfected cells and NES2Y cells. BHK-GIPR transfected cells and NES2Y cells were seeded in 24-well plates with a density of 50,000 and 200,000 cells/well, respectively and incubated overnight at 37 °C. Cells were washed with binding buffer and incubated with approximately 1.6 kBq [Lys^37^(^111^In-DTPA)]N-acetyl-GIP_1-42_ (2.7 fmol) for 1, 2, 4 and 24 h at 37 °C. After incubation, cells were washed twice with binding buffer. To determine the surface-bound fraction, ice-cold acid buffer (0.1 *M* acetic acid, 154 m*M* NaCl, pH 2.5) was added and cells were incubated for 10 minutes at 4 °C. After washing the cells twice with binding buffer to makes sure that all surface-bound tracer was removed from the cells, the cell-associated activity was measured in a well type gamma counter. After removal of the surface-bound activity, the internalized fraction was represented by the remaining cell-associated activity. The internalized fraction (remaining cell-associated activity after acid wash) and the receptor bound fraction (activity removed by acid wash) were determined in a well-type gamma counter.

### Animals

All animal experiments were approved by the Animal Welfare Body of the Radboud University, Nijmegen, The Netherlands and carried out in accordance with their guidelines. Six to eight weeks old, female BALB/c nude mice and C3H mice were purchased from Janvier labs (Le Genest Saint Isle, France). For all *in vivo* experiments, 6-8 weeks old female BALB/c nude mice were injected subcutaneously in the right shoulder with 0.2 ml of a 2.5 × 10^7^ cells/ml suspension of BHK-GIPR transfected cells or with 0.2 ml of a 2.5 × 10^7^ cells/ml suspension of NES2Y cells mixed with matrigel (2:1) in the right shoulder. When the tumor reached a diameter of 2-5 mm, mice were randomly divided in groups.

### Islet Isolation

Islets of Langerhans were isolated from donor C3H mice by collagenase digestion as follows. Mice were euthanized by CO_2_/O_2_ suffocation and 2 ml of ice cold RPMI 1640 containing 1 mg/ml collagenase type V (Sigma Aldrich) were infused via the pancreatic duct *in situ*. After dissection, the perfused pancreata were collected in serum free medium containing collagenase and kept on ice until digestion. Pancreata were digested for 12 min at 37 °C. Digestion was stopped by adding RPMI medium containing 10% FCS, 2 m*M* L-glutamine, 100 U/ml penicillin and 100 U/ml streptomycin (complete RPMI). After washing, digested pancreata were passed through a mesh. Afterwards, islets were purified on a discontinuous Ficoll gradient (densities: 1.108; 1.096; 1.037; Cellgro by Mediatech Inc, Manassas, VA, USA) by centrifugation at 625 × g for 16 min without brake. Islets were collected from the intersection of the second and third layer and remaining Ficoll was removed by washing with complete RPMI. Isolated islets were cultured overnight in complete RPMI medium in a humidified atmosphere containing 5% CO_2_ at 37 °C.

### *In Vitro* Binding to Isolated Islets

After overnight recovery from the isolation procedure, islets from the donor C3H mice were collected, counted and resuspended in complete RPMI. After transwell saturation using binding buffer, islets were transferred to 24 well transwell plates (200 islets per transwell) (Corning Inc. Tewksbury, MA, USA) and washed with binding buffer. Subsequently, approximately 8 kBq [Lys^37^(^111^In-DTPA)]N-acetyl-GIP_1-42_ (13.5 fmol) was added followed by incubation for 24 hours at 37 °C. To investigate the GIPR-mediated binding, 25 µg of unlabeled GIP_1-42_ was added together with [Lys^37^(^111^In-DTPA)]N-acetyl-GIP_1-42_ in a separate set of wells. After incubation, islets were washed extensively with binding buffer and islet-associated radioactivity was measured in a well type gamma counter.

### Peptide Dose Escalation Study

To determine the optimal peptide dose of [Lys^37^(DTPA)]N-acetyl-GIP_1-42_, BHK-GIPR transfected tumor bearing BALB/c nude mice (n = 5/group) were injected intravenously with approximately 3 MBq [Lys^37^(^111^In-DTPA)]N-acetyl-GIP_1-42_ (0.05 µg) in the tail vein and were co-injected with escalating amounts of unlabeled [Lys^37^(DTPA)]N-acetyl-GIP_1-42_ (0.05 µg – 1.95 µg), resulting in final peptide doses ranging from 0.1 to 2 µg/mouse (0.02 – 0.4 nmol/mouse) (5 groups, n = 5/group). Four hours after injection, mice were euthanized by CO_2_/O_2_ suffocation and tumors and other relevant tissues (heart, muscle, lung, spleen, pancreas, kidney, liver, stomach and duodenum) were dissected, weighed and measured in a well type gamma counter. The percentage injected dose per gram of tissue (%ID/g) was determined for each tissue.

### Biodistribution Studies

BALB/c nude mice bearing BHK-GIPR transfected tumors were injected intravenously with approximately 3 MBq (peptide dose 0.2 µg = 0.04 nmol) of [Lys^37^(^111^In-DTPA)]N-acetyl-GIP_1-42_ via the tail vein. At various time points after injection (1, 4 and 24 hours), mice were euthanized by CO_2_/O_2_ suffocation and blood, tumor and other relevant tissues were dissected, weighed and measured in a well type gamma counter. The percentage injected dose per gram (%ID/g) was calculated for each tissue. To determine whether the GIP_1-42_ uptake was GIPR mediated, 25 µg of unlabeled GIP_1-42_ was co-injected in a separate group (n = 5) and mice were euthanized four hours post-injection. BALB/c nude mice bearing NES2Y tumors (n = 5) were injected intravenously with approximately 3 MBq (peptide dose 0.2 µg = 0.04 nmol) of [Lys^37^(^111^In-DTPA)]N-acetyl-GIP_1-42_ via the tail vein. One hour after injection, mice were euthanized by CO_2_/O_2_ suffocation and blood, tumor and other relevant tissues were dissected, weighed and measured in a well type gamma counter. The percentage injected dose per gram (%ID/g) was determined for each tissue. To determine whether the GIP_1-42_ uptake was GIPR mediated, 25 µg of unlabeled GIP_1-42_ was co-injected in a separate group (n = 2) and mice were euthanized one hours post-injection.

### SPECT/CT

One (BHK-GIPR tumors and NES2Y tumors) or four (BHK-GIPR tumors)hours (n = 2/group) prior to SPECT imaging, BALB/c nude mice were injected intravenously in the tail vein with 20.6 ± 0.7 MBq [Lys^37^(^111^In-DTPA)]N-acetyl-GIP_1-42_ (peptide dose 0.2 µg ( = 0.04 nmol) in 200 µl injection fluid). SPECT/CT scans were acquired on a dedicated small animal SPECT/CT scanner (U-SPECT-II, Milabs, Utrecht, The Netherlands) with a 1.0 mm mouse collimator, using 36 bed positions and an acquisition time of 30 minutes (BHK-GIPR tumors) or 45 minutes (NES2Y tumors). After SPECT acquisition, CT (65 kV, 615 µA, 1 bed position) was acquired as an anatomical reference. SPECT scans were reconstructed using U-SPECT-II reconstruction software (U-SPECT-Rec, Milabs, Utrecht, The Netherlands) with the following settings: selection of the lower ^111^In photopeak (152–183 keV), corrected for two backgrounds (135–151 keV and 184–211 keV), pixel based OSEM, voxel size 0.4 mm^3^ and 1 iteration over 16 subsets.

### Statistics

All values are expressed as mean ± standard deviation (SD). Statistical analysis was performed using unpaired two-tailed t-test using GraphPad Prism v5.03 (GraphPad Softwarem Inc., San Diego, CA, USA). The level of significance was set at p < 0.05.

### Ethical approval

All animal experiments were approved by the Animal Welfare Body of the Radboud University, Nijmegen, The Netherlands and carried out in accordance with their guidelines.

### Data availability statement

all data are available at the department of Nuclear medicine of the Radboud University medical center, Nijmegen.

## Electronic supplementary material

Raw data of biodistribution studies
