# Supplementary material for: Characterization of 111In-labeled Glucose-Dependent Insulinotropic Polypeptide as a Radiotracer for Neuroendocrine Tumors
Source: Sci Rep. 2018 Feb 13;8:2948. doi: 10.1038/s41598-018-21259-3 (PMC5811606; doi:10.1038/s41598-018-21259-3)
Supplement: Supplementary file 1 — Raw data of biodistribution studies [file 41598_2018_21259_MOESM1_ESM.docx]

**Characterization of ^111^In-labeled Glucose-Dependent Insulinotropic Polypeptide as a Radiotracer for Neuroendocrine Tumors**

Stefanie M.A. Willekens^1,2*+^, Lieke Joosten^1+^, Otto C. Boerman^1^, Maarten Brom^1^, Martin Gotthardt^1^

^+^These authors contributed equally

^1^Department of Radiology and Nuclear Medicine, Radboud university medical center, Nijmegen, The Netherlands

^2^Division of Nuclear Medicine, Department of Imaging and Pathology, University Hospitals and KU Leuven, Belgium

**Supplementary Tables**

**Supplementary table 1:** Raw data of the peptide dose escalation study in BALB/c nude mice with subcutaneous BHK-GIPR tumors shown in figure 5a. Values are expressed as percentage injected dose per gram tissue (%ID/g).

|  | 0.1 µg (n=5) | | 0.2 µg (n=5) | | 0.5 µg (n=5) | | 1 µg (n=5) | | 2 µg (n=5) | |
| --- | --- | --- | --- | --- | --- | --- | --- | --- | --- | --- |
|  | **mean** | **SD** | **mean** | **SD** | **mean** | **SD** | **mean** | **SD** | **mean** | **SD** |
| Blood | 0.033 | 0.007 | 0.047 | 0.010 | 0.047 | 0.009 | 0.059 | 0.015 | 0.066 | 0.047 |
| Tumor | 0.146 | 0.032 | 0.125 | 0.023 | 0.135 | 0.047 | 0.143 | 0.032 | 0.163 | 0.032 |
| Muscle | 4.692 | 0.845 | 5.185 | 1.363 | 2.358 | 0.672 | 2.880 | 0.340 | 1.921 | 0.085 |
| Heart | 0.133 | 0.029 | 0.156 | 0.030 | 0.163 | 0.016 | 0.178 | 0.023 | 0.173 | 0.020 |
| Lung | 0.364 | 0.067 | 0.518 | 0.117 | 0.577 | 0.192 | 0.608 | 0.082 | 0.560 | 0.130 |
| Spleen | 0.648 | 0.096 | 0.692 | 0.176 | 0.594 | 0.151 | 0.781 | 0.076 | 0.713 | 0.066 |
| Pancreas | 0.169 | 0.027 | 0.234 | 0.051 | 0.199 | 0.051 | 0.257 | 0.035 | 0.213 | 0.035 |
| Kidney | 237.3 | 17.75 | 265 | 38.01 | 213.8 | 41.68 | 287.25 | 33.58 | 256.96 | 28.78 |
| Liver | 1.604 | 0.308 | 1.533 | 0.320 | 1.550 | 0.371 | 1.738 | 0.243 | 1.831 | 0.352 |
| Stomach | 0.410 | 0.092 | 0.418 | 0.092 | 0.411 | 0.031 | 0.527 | 0.101 | 0.437 | 0.069 |
| Duodenum | 0.486 | 0.100 | 0.573 | 0.151 | 0.529 | 0.064 | 0.750 | 0.270 | 0.929 | 0.658 |
| Tumor/Blood | 143.4 | 15.75 | 109.8 | 13.06 | 52.552 | 18.371 | 50.276 | 7.419 | 37.95 | 16.3 |
| Tumor/Muscle | 33.69 | 10.64 | 41.12 | 5.161 | 19.08 | 8.460 | 20.94 | 5.020 | 12.23 | 2.944 |
| Tumor/Kidney | 0.020 | 0.005 | 0.019 | 0.003 | 0.011 | 0.002 | 0.01 | 0.001 | 0.008 | 0.001 |
| Tumor/Heart | 35.64 | 2.049 | 33.10 | 5.330 | 14.58 | 4.15 | 16.45 | 3.023 | 11.22 | 1.417 |
| Tumor/Lung | 12.96 | 1.162 | 10.04 | 1.817 | 4.185 | 0.722 | 4.807 | 0.823 | 3.575 | 0.825 |

**Supplementary table 2:** Raw data of the biodistribution study of [Lys^37^(^111^In-DTPA)]N-acetyl-GIP_1-42_ in BALB/c nude mice bearing subcutaneous NES2Y tumors shown in figure 6. Values are expressed as percentage injected dose per gram tissue (%ID/g).

|  | NES2Y 1h pi (n=5) | | NES2Y 1h pi + xs (n=5) | | Statistics | | |
| --- | --- | --- | --- | --- | --- | --- | --- |
|  | **mean** | **SD** | **mean** | **SD** | | **p-value** | **significant** |
| Blood | 0.175 | 0.072 | 0.214 | 0.058 | |  |  |
| Tumor | 0.653 | 0.160 | 0.619 | 0.014 | | 0.700 | no |
| Muscle | 0.069 | 0.012 | 0.057 | 0.01 | |  |  |
| Heart | 0.135 | 0.018 | 0.103 | 0.017 | |  |  |
| Lung | 0.485 | 0.041 | 0.474 | 0.077 | |  |  |
| Spleen | 0.462 | 0.122 | 0.341 | 0.013 | |  |  |
| Pancreas | 0.201 | 0.036 | 0.158 | 0.037 | | 0.200 | no |
| Kidney | 184.38 | 17.56 | 180.17 | 19.22 | |  |  |
| Liver | 1.105 | 0.113 | 0.827 | 0.123 | |  |  |
| Stomach | 0.336 | 0.062 | 0.283 | 0.038 | |  |  |
| Duodenum | 0.42 | 0.122 | 0.369 | 0.042 | |  |  |
| Tumor/Blood | 3.959 | 0.758 | 3.013 | 0.878 | |  |  |
| Tumor/Muscle | 9.425 | 1.121 | 11.09 | 2.051 | |  |  |
| Tumor/Kidney | 0.004 | 0.001 | 0.004 | 0.0004 | |  |  |
| Tumor/Heart | 4.825 | 0.961 | 6.092 | 1.141 | |  |  |
| Tumor/Lung | 1.376 | 0.448 | 1.326 | 0.243 | |  |  |

**Supplementary table 3:** Raw data of the biodistribution study of [Lys^37^(^111^In-DTPA)]N-acetyl-GIP_1-42_ in BALB/c nude mice bearing subcutaneous BHK-GIPR tumors at 1, 4 and 24 hours after tracer injection shown in figure 6a. Values are expressed as percentage injected dose per gram tissue (%ID/g).

|  | BHK-GIPR 1h pi (n=5) | | BHK-GIPR  4h pi (n=5) | | BHK-GIPR 4h pi + xs (n=5) | | BHK-GIPR  24h pi (n=5) | | Statistics | |
| --- | --- | --- | --- | --- | --- | --- | --- | --- | --- | --- |
|  | **mean** | **SD** | **mean** | **SD** | **mean** | **SD** | **mean** | **SD** | **p** | **signif** |
| Blood | 0.248 | 0.121 | 0.045 | 0.015 | 0.031 | 0.010 | 0.017 | 0.006 |  |  |
| Tumor | 4.694 | 0.753 | 3.267 | 0.772 | 0.855 | 0.202 | 1.946 | 0.213 | 0.0001 | yes |
| Muscle | 0.135 | 0.020 | 0.082 | 0.007 | 0.068 | 0.012 | 0.058 | 0.019 |  |  |
| Heart | 0.200 | 0.030 | 0.116 | 0.016 | 0.096 | 0.020 | 0.081 | 0.027 |  |  |
| Lung | 0.545 | 0.083 | 0.332 | 0.076 | 0.331 | 0.111 | 0.184 | 0.059 |  |  |
| Spleen | 0.603 | 0.089 | 0.528 | 0.173 | 0.519 | 0.124 | 0.493 | 0.076 |  |  |
| Pancreas | 0.296 | 0.036 | 0.193 | 0.024 | 0.147 | 0.019 | 0.137 | 0.026 | 0.01 | yes |
| Kidney | 210.9 | 22.93 | 227.9 | 15.46 | 227.1 | 25.814 | 172.75 | 21.28 |  |  |
| Liver | 1.100 | 0.147 | 1.155 | 0.259 | 1.094 | 0.266 | 0.695 | 0.160 |  |  |
| Stomach | 0.502 | 0.115 | 0.362 | 0.069 | 0.268 | 0.024 | 0.258 | 0.067 |  |  |
| Duodenum | 0.561 | 0.085 | 0.448 | 0.045 | 0.392 | 0.068 | 0.378 | 0.043 |  |  |
| Tumor/Blood | 21.76 | 7.926 | 77.6 | 21.65 | 29.76 | 10.10 | 129.79 | 68.35 |  |  |
| Tumor/Muscle | 34.97 | 4.702 | 40.43 | 11.33 | 12.45 | 1.14 | 36.351 | 11.38 |  |  |
| Tumor/Kidney | 0.023 | 0.006 | 0.014 | 0.004 | 0.004 | 0.001 | 0.011 | 0.001 |  |  |
| Tumor/Heart | 23.58 | 3.414 | 29 | 9.682 | 8.894 | 0.831 | 25.505 | 5.307 |  |  |
| Tumor/Lung | 8.640 | 1.053 | 10.09 | 2.907 | 2.680 | 0.510 | 11.271 | 2.954 |  |  |
